# Malignant Peripheral Nerve Sheath Tumors of the Head and Neck: A Case Series and Literature Review

**DOI:** 10.1155/2014/368920

**Published:** 2014-12-04

**Authors:** Brandon T. Mullins, Trevor Hackman

**Affiliations:** ^1^University of North Carolina School of Medicine, 1033 Bondurant Hall, Chapel Hill, NC 27514, USA; ^2^Division of Otolaryngology, University of North Carolina Hospitals, 101 Manning Drive, Chapel Hill, NC 27514, USA

## Abstract

*Background*. Malignant peripheral nerve sheath tumors (MPNSTs) of the head and neck are rare aggressive neoplasms with a poor prognosis. This study describes the management and outcomes of 3 of our patients with MPNSTs of the head and neck. *Methods*. We identified 3 patients presenting with MPNST of the head and neck and treated at the University of North Carolina. We compared our results to the literature from 1963 to 2014. *Results*. Mean follow-up was 31 months. Average age at diagnosis was 44.7 years of age. All patients received wide-local excision and adjuvant radiotherapy. No patients recurred during the series. Recurrence-free survival time for the patients was 45, 37, and 3 months, respectively. *Conclusions*. Our data series confirms that a combined-modality approach with complete surgical resection and adjuvant radiotherapy leads to improved outcomes in MPNSTs of the head and neck. Nonetheless, due to historically poor outcomes, continued research into newer therapies needs to be explored.

## 1. Introduction

Malignant peripheral nerve sheath tumors (MPNSTs) are rare high-grade sarcomas arising from peripheral nerves or cells of the peripheral nerve sheath [1, 2]. They account for approximately 5–10% of all soft-tissue sarcomas, with only about 8–16% occurring in the head and neck region [2–7]. They are commonly associated with neurofibromatosis type 1 (NF1), an autosomal dominant disorder resulting from the defective tumor suppressor protein neurofibromin, but can also arise through sporadic mutation [7, 8]. Based on historically high rates of local recurrence and rapid disease progression, the prognosis for MPNSTs is generally poor despite aggressive therapy [7, 8].

This IRB-approved retrospective case series describes three patients with MPNSTs of the head and neck treated at the University of North Carolina. They were diagnosed based on a thorough history and physical examination and confirmed through biopsy and histopathological analysis. For all three cases, the treatment method consisted of a combined-modality approach of wide-local surgical excision and adjuvant radiation therapy to aid in local control. Our goal is to add to the literature by reporting the clinical presentation, prognostic factors, and outcomes from our single institutional study with a literature review.

## 2. Clinical Cases

### 2.1. Case 1

*History.* A 46-year-old female with a previous history of multiple sclerosis was admitted to our hospital for two months of nasal stuffiness, rhinorrhea, and severe headaches. The past history was otherwise unremarkable.

**Figure 1:**
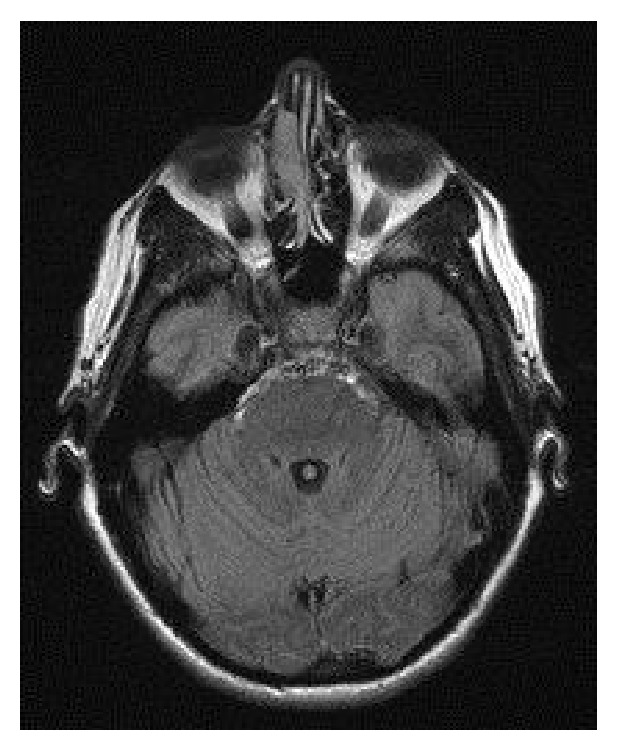
T1-weighted axial MRI demonstrating the mass in the right nasal cavity.

**Figure 2:**
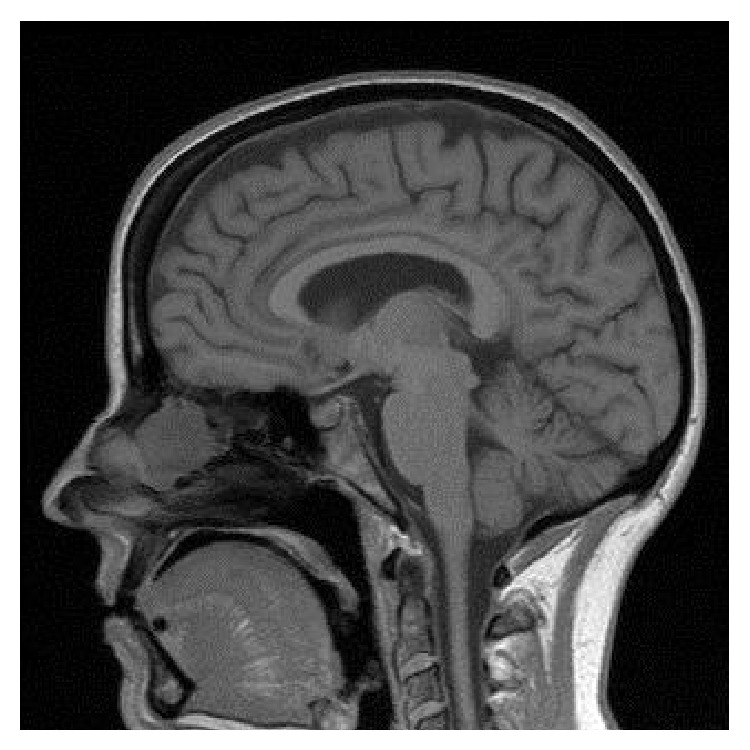
T1-weighted sagittal MRI of the nasal cavity mass.

*Examination.* Head and neck physical examination was unremarkable without evidence of facial tenderness, masses, lesions, or palpable adenopathy. Endoscopy was then performed for further assessment. Sinonasal endoscopy revealed a large polypoid appearing mass of the right nasal cavity occupying a significant portion of the anterior nasal cavity and extending inferiorly to the mid portion of the inferior turbinate. There was no purulence or clear rhinorrhea appreciated with Valsalva. Normal anatomy was appreciated in the left nasal cavity. A subsequent MRI revealed a 3.3 × 1.1 cm enhancing soft-tissue mass, hypointense on T1 and hyperintense on T2, in the anterior nasal cavity, medial to the middle turbinate, seeming to originate from the anterior skull base (Figures 1 and 2).

*Management.* The skull base mass was excised through an endonasal resection including a right ethmoidectomy, mucus membrane removal within the right sphenoid and maxillary sinuses, and middle turbinate resection to the level of the skull base. During surgery, the tumor was found to involve the right aspect of the cribriform plate at the base of the skull. Pathologic examination of the specimen revealed a 0.7 cm grade 1 spindle-cell tumor with features consistent with MPNST, unfortunately resected with positive margins. Immunohistochemical analysis of the tumor revealed reactivity for S100 and vimentin, with no reactivity for muscle specific actin, smooth muscle actin, myogenin, desmin, EMA, PR, CD34, HMB45, Mart-1, and cytokeratins. A repeat excision with negative margins was then performed using an intradural craniofacial approach to the anterior cranial fossa using an endoscopic endonasal technique. Intradural resection of the anterior cranial fossa was performed in addition to bilateral ethmoidectomies, bilateral maxillary antrostomies and sphenoidotomy with tissue removal, bilateral frontal sinusotomy, and a left nasoseptal flap reconstruction. Based on the potential for microscopic disease and the difficulty of obtaining clear margins in this region, adjuvant radiation therapy (60 Gy) was recommended and carried out at an outside institution. The patient was followed up with no major complications related to surgery or radiation therapy. She was recurrence-free for 45 months at last follow-up.

### 2.2. Case 2

*History.* A 55-year-old female with a previous history of Charcot-Marie-Tooth disease presented to our institution with a three-month history of dizziness and vertigo, periorbital, frontal, and left cheek soreness, worsening diplopia, and weight loss. The past history was otherwise unremarkable. 

**Figure 3:**
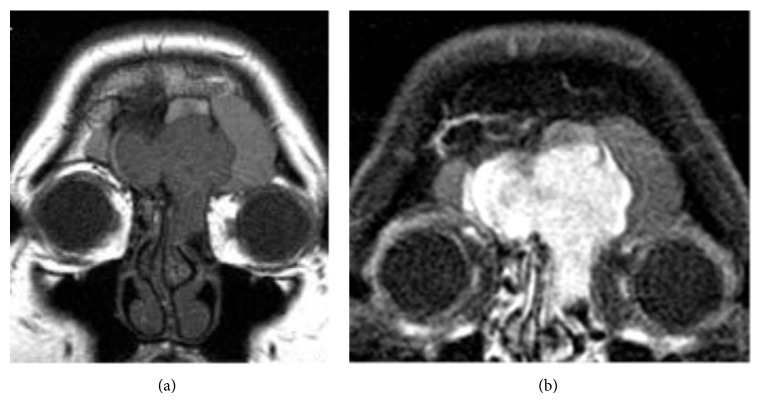
((a) and (b)) Preoperative coronal MRI images showing the tumor's frontal sinus location and (b) demonstrating tumor's strong contrast enhancement.

**Figure 4:**
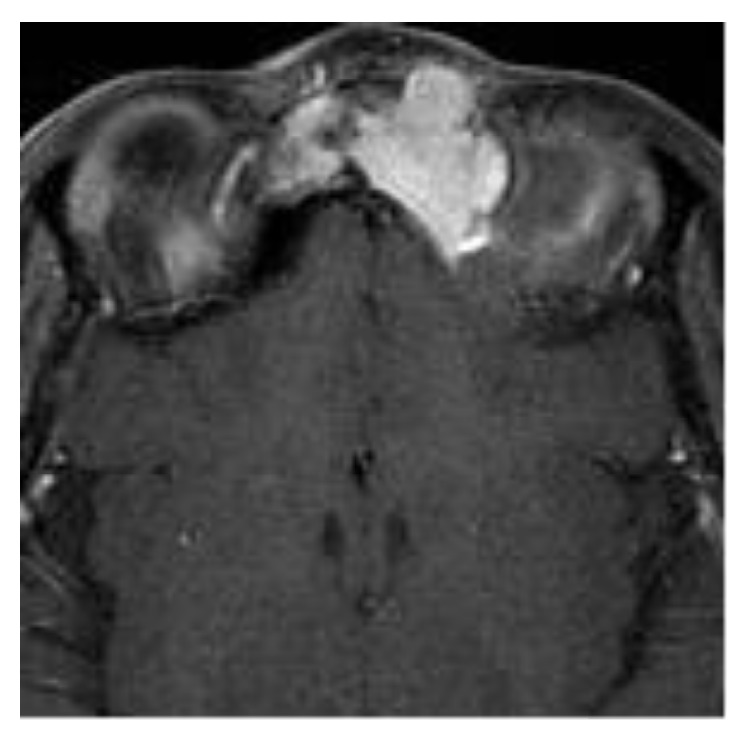
Preoperative axial MRI image of the tumor.

*Examination.* Physical examination revealed left eye pseudoptosis and 3-4 mm of proptosis, an asymmetric pupillary light reflex, subjective diplopia, and tender frontal and left maxillary sinuses. To better evaluate the patient's symptoms, sinonasal endoscopy was performed revealing a hyperemic mass in the anterior ethmoids in the left nasal cavity. Subsequent MRI revealed a 5.7 × 2.1 cm diffusely enhancing soft-tissue mass centered in the left frontal sinus extending into the left anterior ethmoid air cells. The mass showed multifocal bony dehiscence of the frontal sinuses and left frontal recess with extension into the superior left orbit abutting the superior rectus muscle and globe (Figures 3 and 4). There was also pathologic ophthalmic nerve involvement. Biopsy was performed revealing a malignant spindle-cell neoplasm with interlacing fascicles and moderate nuclear pleomorphism, most consistent with a malignant peripheral nerve sheath tumor. Immunohistochemical analysis revealed S-100, CD99, and TLE1 reactivity with no reactivity for cytokeratins, desmin, Mart-1, HMB56, CD34, SMA, EMA, or GFAP. Distant metastasis was ruled out by 18F-fluorodeoxyglucose positron emission tomography. 

**Figure 5:**
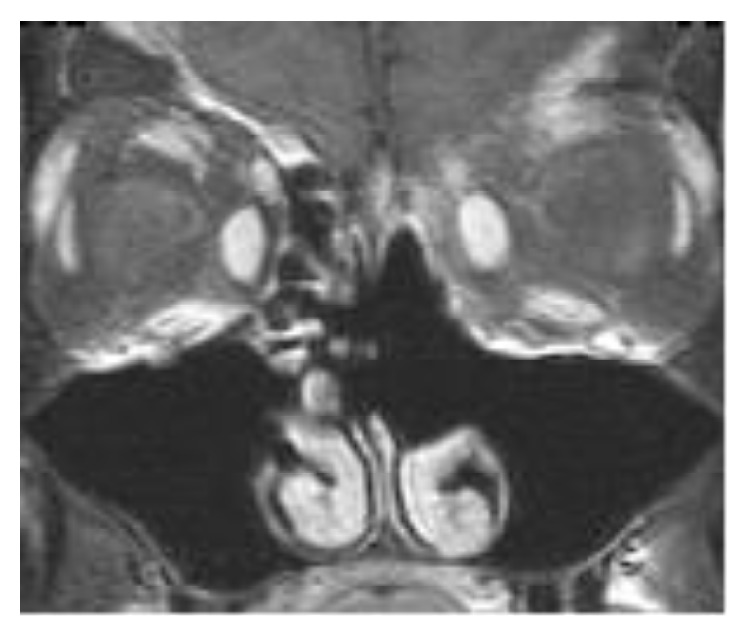
Coronal MRI 2 years after operation showing no residual or recurrent tumor.

*Management.* The frontal mass was excised with negative margins through a craniofacial approach. Procedure involved a coronal incision for bilateral craniotomy for extradural resection of the frontal sinus-based mass including a left ethmoidectomy partial sphenoidotomy and left maxillary antrostomy with tissue removal. Complete closure was then performed using a pericranial flap and abdominal fat graft. Pathologic examination was similar to the biopsy specimen and confirmed the presence of a 4.5 cm grade 2 MPNST. Due to close margins at the cribriform plate, adjuvant radiation therapy was recommended to improve local control. One month after surgical resection, five weeks of radiation therapy was delivered to the frontal and ethmoid sinuses at a cumulative dose of 69 Gy. The treatment was well tolerated; complete remission was achieved; and at last follow-up she had been recurrence-free for 37 months (Figure 5).

### 2.3. Case 3

*History.* A 34-year-old male with a known three-year history of NF1, characterized by numerous central and peripheral plexiform neurofibromas, was admitted for airway obstruction due to a large left parapharyngeal/carotid space mass. The patient was known at our institution as he presented with a MPNST of the left lower extremity three years before and treated with neoadjuvant radiation therapy and surgical resection leading to complete remission. Based on his history of NF1 and MPNST, this parapharyngeal space mass was suspected to be malignant degeneration of a neurofibroma, similar to his prior MPNST. 

**Figure 6:**
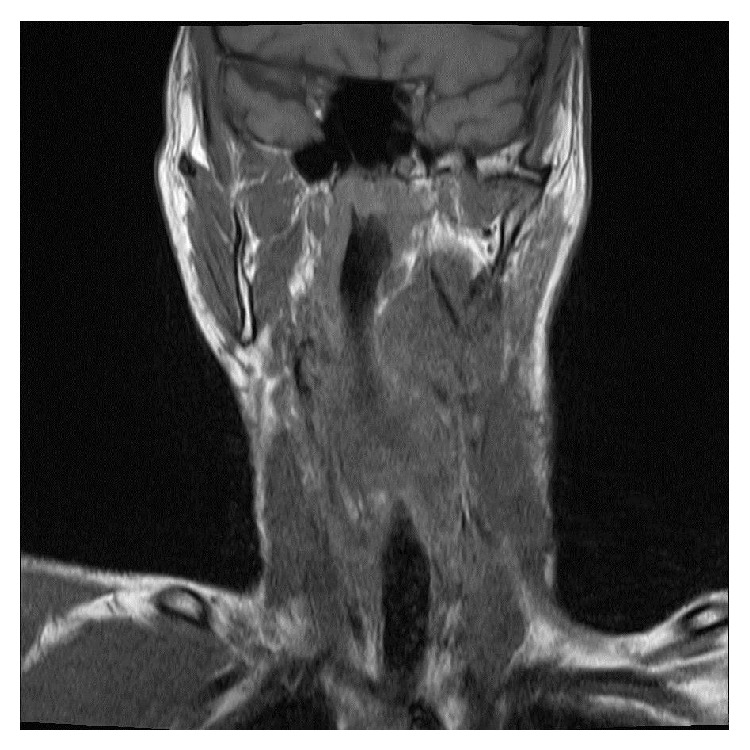
Coronal T1-weighted MRI showing the left pharyngeal mass with airway depression.

*Examination.* MRI evaluation increased this suspicion, showing a 5.2 × 3.3 × 4.9 cm well-defined, heterogeneous mass (isointense to hyperintense on T1 and hyperintense on T2) depressing the oropharyngeal supraglottic airway (Figure 6) in addition to innumerable other neurofibromas and schwannomas. The mass appeared to extend along the hypoglossal nerve into the hypoglossal foramen and encasing the carotid artery. Due to surgical urgency of airway compromise, no presurgical biopsy was performed. 

*Management.* The parapharyngeal space mass was then excised with positive margins through a left parapharyngeal space resection and a left selective neck dissection (levels 2 and 3). The mass was noted to emanate from either the vagus or the hypoglossal nerve and encase the carotid artery at the bifurcation; the vagus nerve was ultimately sacrificed. Pathology confirmed the presence of a 6.3 × 4.7 × 3.5 cm grade 2 MPNST arising from a preexistent neurofibroma. Lymph nodes were negative for malignancy. Adjuvant radiation therapy was recommended after surgery in order to increase the rate of local control. Unfortunately, the surgery was complicated by a left middle cerebral artery ischemic stroke leading to right spastic hemiplegia and aphasia. The patient underwent intensive inpatient rehabilitation with seven weeks of concurrent radiation therapy to a cumulative dosage of 70 Gy. Radiation therapy was well-tolerated, and patient had been recurrence-free for 3 months (postsurgical resection) at last follow-up.

## 3. Discussion

MPNSTs are very rare tumors with incidents of approximately 0.001% in the population [3, 9]. These tumors usually affect the proximal extremities and trunk and are very rare in the head and neck [6, 10–12]. They usually arise in adulthood, with most occurring around 20–50 years of age [13]. MPNSTs can occur sporadically, as seen in our cases 1 and 2, or more commonly as a consequence of malignant degeneration of a neurofibroma in patients with NF1, as seen in case 3. For patients with NF1, the lifetime risk of developing a MPNST is much higher at approximately 10% [12, 14].

Clinically, these tumors usually present as an enlarging palpable mass with or without pain [15, 16]. Based on the internal nature of our patient's head and neck MPNSTs, the presenting symptoms (severe headaches, diplopia, airway obstruction, etc.) are related to the tumor's anatomic location as it enlarged (Table 1). Rapid tumor enlargement is more common in patients with NF1-associated MPNSTs [17, 18], which could relate to the suddenness in which our NF1 patient in case 3 presented with airway obstruction.

Similar to most soft-tissue sarcomas, surgery is the primary modality used to treat MPNSTs of the head and neck, with complete surgical excision of the tumor with negative margins correlating with longer overall survival [2, 6, 7, 18–21]. However, given the relatively small space within the head and neck, the proximity to vital structures, and the potential for microscopic local spread, obtaining negative margins can be difficult. These factors have led to high rates of local recurrence (22% to 52%) and distant metastasis (18% to 33%) and thus a multimodality treatment approach is most favorable [2, 22–24].

In addition to surgery, many studies have cited the importance of radiotherapy as adjunctive treatment, especially for improving local control [2, 15, 18, 20, 24–27]. The radiation dose commonly administered in studies is 50–60 Gy. The oncology consensus group, as part of a uniform treatment policy for MPNSTs, recommends adjuvant radiotherapy for all “intermediate- to high-grade lesions and for low-grade tumors after a marginal excision” [18]. Though the consensus group states that radiotherapy only provides local control and has little effect on long-term survival, some more recent studies have also reported survival benefit [2, 15, 24, 28]. Our series continues to highlight the importance of adjunctive radiotherapy. In our series, all three patients underwent adjuvant radiotherapy after their surgical resection. These three patients, despite margin status, have remained recurrence-free using this combined treatment approach (Table 2). Of note, the radiation doses delivered were slightly higher than commonly reported in the literature (our mean was 66 Gy) which may have contributed to the increased local control rates, though this is purely speculative.

Studies have also looked at the benefit of chemotherapy in the treatment of MPNSTs, though the conclusions have been controversial [29]. While none of our patients underwent chemotherapy, some studies have shown benefit in survival and prevention of local and distant recurrence [30, 31]. Moretti et al. reported a 2-year overall survival rate of 80% and disease-free survival of 57% when using doxorubicin and ifosfamide in combination with surgery and radiation therapy in 10 patients with MPNSTs [30]. As well, Schuetze and Patel reported that chemotherapy, either adjuvant or neoadjuvant, should be considered for patients with large (>5 cm) and high-grade MPNSTs, regardless of location. They reported that the preferred chemotherapy regimen is a dose-intensive anthracycline and ifosfamide combination with growth factor support [31]. However, a recent study by Kolberg et al. reported that their patients who were selected for chemotherapy seemed to have a worse prognosis and lower survival rates [32]. Unfortunately, this lack of significant improvement in survival has also been shown by many other studies [9, 16, 18, 20, 21, 27, 33, 34]. Nonetheless, chemotherapy may be helpful for local disease control for marginally resected head and neck MPNSTs at sites in which adequate radiotherapy is difficult to deliver [18].

Based on the rarity of head and neck MPNSTs, survival and prognostic data in the literature is mostly limited to case reports and smaller series. In the literature of the past 10 years, the 5-year overall survival and disease-specific survival rates for head and neck MPNSTs have ranged from approximately 28% to 40% and from 20% to 44%, respectively [2, 21, 23, 24, 35, 36]. In analyzing MPNSTs from all body sites, most studies agree that the significant prognostic factors for survival include surgical margin status and tumor size [2, 9, 20, 21, 27, 32, 37, 38]. Some also report that histologic grade is significant with high-grade status relating to a poorer prognosis [15, 20, 27, 32, 37]. A more controversial topic is whether NF1 status significantly affects prognosis, as there have been conflicting reports. Evans et al. and Sordillo et al. found 5-year overall survival rate differences of approximately 20% between their patients with NF1-associated MPNSTs compared to sporadic cases, with the NF1-associated group having the poorer rates [14, 34]. As overall survival rates for MPNSTs have improved with time, there has also been more convergence reported in these rates. In more recent studies, Anghileri et al. and Lafemina et al. both reported no significant difference in the survival rate of their patients with NF1-associated MPNSTs compared to their sporadic MPNST patients [2, 38]. A recent large survival meta-analysis by Kolberg et al. also came to the same conclusion [32]. This convergence in rate over time could be due to improved early recognition and diagnosis of MPNSTs in NF1 patients as well as improved treatment strategies overall.

Our case series describes significantly better outcomes than those reported in the literature. Despite the poor local recurrence and survival rates previously reported, our patients in cases 1 and 2 have survived recurrence-free for 45 and 37 months posttreatment, respectively. Our patient in case 3 has also yet to show recurrence while undergoing their adjuvant radiation therapy. These high rates of recurrence-free survival are most likely due to obtaining negative surgical margins (66.7% of our cases) as well as utilizing adjuvant radiotherapy. As stated previously, the increased radiation treatment dose delivered may also have contributed to decreased recurrence rates. While we do not yet have 5-year post-treatment results, these recurrence and survival rates at 3 years post-treatment are promising results.

## 4. Conclusion

This case series reports on three instances of MPNSTs of the head and neck. Since they are very rare neoplasms, literature remains sparse on the prognostic factors and ideal treatment methods to control these tumors. While surgical excision with negative margins and radiation therapy seems to be the current mainstay, the prognosis historically remains poor. Nonetheless, careful follow-up in patients with NF1 and timely diagnosis and treatment is necessary. Research into new therapies, including possible molecularly targeted therapeutics, needs further study in order to continue to improve local control and survival rates.

## Figures and Tables

**Table 1:** Clinical characteristics of the MPNST series.

Case	Age	Gender	Location	Pathologic tumor size	Symptoms
1	46	Female	Skull base	0.7 cm	Nasal stuffiness, rhinorrhea, and severe headaches
2	54	Female	Frontal sinus	4.5 cm	Facial soreness, vertigo, diplopia, and weight loss
3	34	Male	Parapharyngeal/carotid space	6.3 cm	Airway obstruction

**Table 2:** Treatment and outcomes of the MPNST series.

Case	Treatment	Margin status	Radiation dosage	Recurrence	Recurrence-free survival time
1	Surgery + adjuvant radiotherapy	Negative	60 Gy	None	45 months
2	Surgery + adjuvant radiotherapy	Negative	69 Gy	None	37 months
3	Surgery + adjuvant radiotherapy	Positive	70 Gy	None	3 months (after surgery)
